# Management of an Aortoesophageal Fistula With Esophageal Endoluminal Wound Vacuum Therapy

**DOI:** 10.1016/j.atssr.2024.01.011

**Published:** 2024-02-15

**Authors:** Antoine Nehme, Samuel Brown, Salman Zaheer, Alexander Leung

**Affiliations:** 1 Department of Cardiothoracic Surgery, Loma Linda University Health, Loma Linda, California

## Abstract

A 39-year-old man with past medical history of type A aortic dissection presented to the emergency department with hematemesis, hypotension, and tachycardia. Imaging revealed an aortoesophageal fistula. The patient was taken emergently for thoracic endovascular aortic repair to cover the area of potential fistula. Due to the patient being a poor operative candidate, the decision was made to treat with endoluminal esophageal wound vacuum therapy. He underwent twice weekly endoscopies with sponge changes until discharge; he has done well since. Wound vacuum therapy in conjunction with thoracic endovascular aortic repair may represent a treatment option for patients with aortoesophageal fistula who are poor candidates for surgery.

Aortoesophageal fistula (AEF) is a rare but complex and morbid late complication after aortic surgery. AEFs most often arise in those with a history of aortic surgery; however, they can also be seen in those with aortic aneurysms and, less commonly, in patients with thoracic malignancies, foreign body ingestion, esophageal trauma, and tuberculosis. Traditional open operative repair requires a reoperative surgery involving resection of the aortic graft and then either replacement or an extraanatomic aortic reconstruction. In addition, the esophageal perforation must be addressed by either a primary repair or esophagectomy with reconstruction. As endovascular therapies have advanced, there have been some reports of using thoracic endovascular aortic repair (TEVAR) to stent the aorta.^1^^,^^2^

Endoluminal wound vacuum (E-Vac) therapy is a relatively new tool that can be used to treat esophageal perforations. There have been reports of using E-Vac therapy to treat esophageal perforations and fistulas with good success, but it has not been used to treat AEF.^3^^,^^4^ We present a case in which a 39-year-old male patient with an aortoesophageal fistula was managed with TEVAR followed by E-vac therapy.

A 39-year-old man with a past medical history of Stanford type A aortic dissection at age 14 years who underwent ascending aorta and hemiarch replacement, followed by redo sternotomy and aortic arch repair for aneurysm of the aortic arch, and then left thoracotomy for repair of a proximal descending thoracic aortic aneurysm, presented to the Loma Linda University Medical Center emergency department with 1 day of massive hematemesis, hypotension, tachycardia, and fevers. He had been experiencing chest pain, fatigue, and cough for around 3 months before he began experiencing hematemesis. This patient most likely has a connective tissue disorder, but never received genetic testing to confirm such. A computed tomography angiogram of his chest was obtained, which was concerning for aortoesophageal fistula (Figure 1). Laboratory studies showed leukocytosis and lactic acidosis, elevated erythrocyte sedimentation rate and C-reactive protein, and normal creatinine. The patient was started on empiric broad antibiotics and taken emergently to the operating room for TEVAR with a Cook 38-34 (tapered) x 154 mm Zenith TX2 dissection endovascular graft with intravascular ultrasound guidance. An esophagogastroduodenoscopy was then performed after the TEVAR, which revealed a proximal esophageal diverticulum with erosion of the wall and visible descending thoracic aortic graft at 25 cm from the incisors (Figure 2). There was purulent drainage coming from this space into the esophageal lumen. The patient had a poor nutritional status with an albumin level of 2.0. Given the high risk of open reoperative surgery to reconstruct his descending thoracic aorta and address the esophageal perforation, the decision was made to treat his esophageal perforation with endoluminal esophageal wound vacuum therapy. E-Vac therapy was initiated using a technique similar to that described by Leeds and associates.^5^ Suction was set to 125 mm Hg for the duration of therapy. A percutaneous endoscopic gastrostomy tube had been placed for enteric feeding access during this time. The patient was made nil per os and underwent twice weekly E-Vac sponge changes. During this period, the patient’s esophageal wall defect progressively decreased in size and the potential space adjacent to the aortic graft was obliterated. The total duration of E-Vac therapy was 4 weeks. The patient underwent repeat imaging that showed no remaining evidence of esophageal fistula (Supplemental Figure 1). He was transitioned to enteric antibiotics with amoxicillin-clavulanate and ultimately discharged on hospital day 51. A follow-up endoscopy 4 months after discharge revealed a well-healed ulcer at the location of the fistula that was completely covered by mucosa (Figure 3). The patient was eventually transitioned back to an oral diet, and antibiotics were discontinued 8 months after discharge at the recommendation from the infectious disease team; all cultures remained negative. The patient continues to do well at 17 months post-discharge.

**Figure 1:**
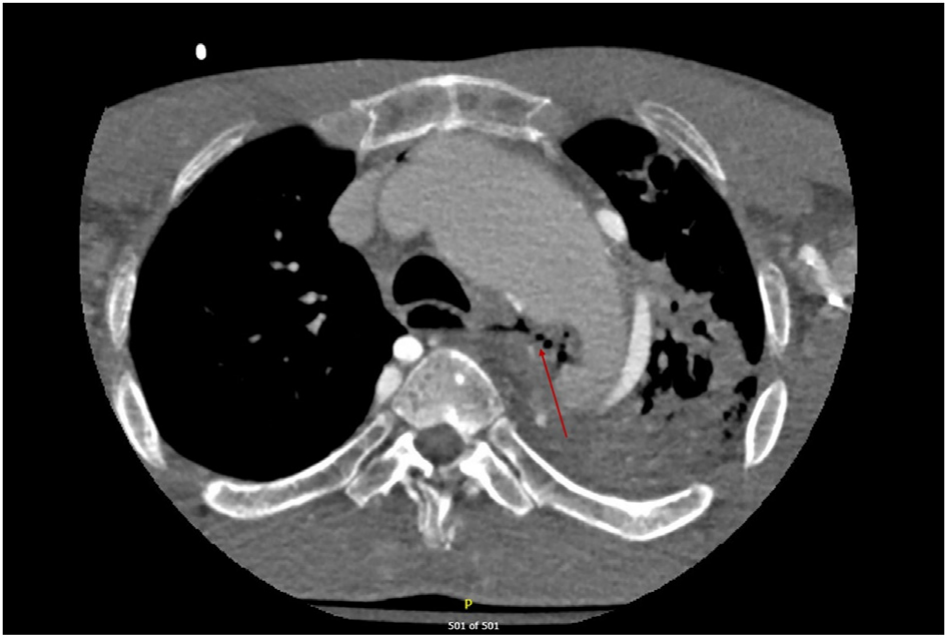
Computed tomography scan of the patient’s chest shortly after presentation to the emergency department. Red arrow shows gas tracking from esophagus to aorta concerning for aortoesophageal fistula. Left upper lobe consolidation visible at bottom right of image.

**Figure 2:**
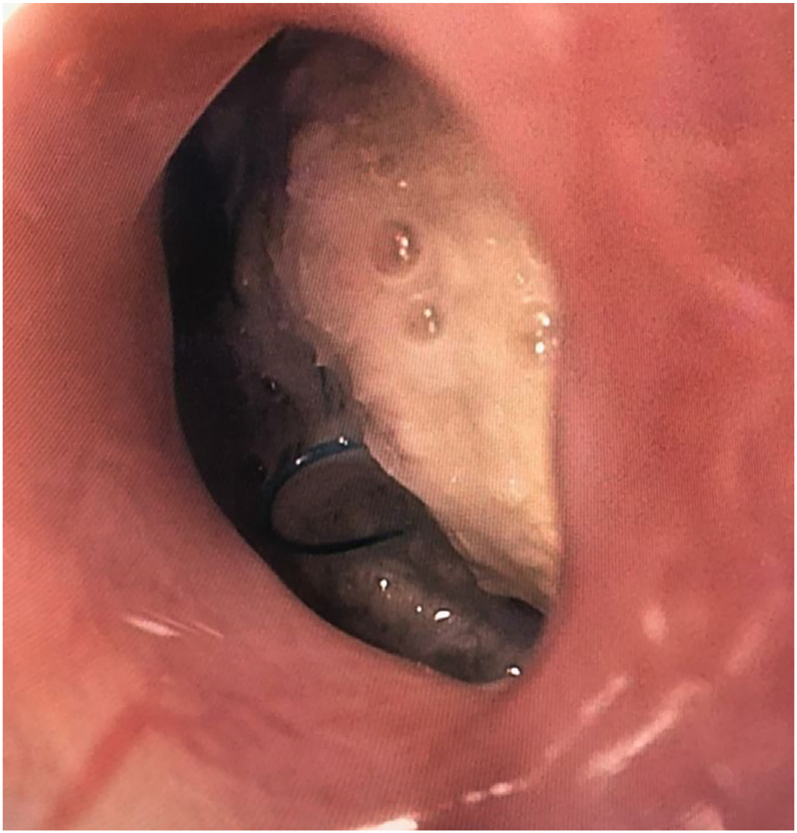
Esophagogastroduodenoscopy prior to wound vacuum therapy showing esophageal defect. Aortic graft can be seen through the defect, blue prolene suture is visible at the bottom left area of the defect.

**Figure 3:**
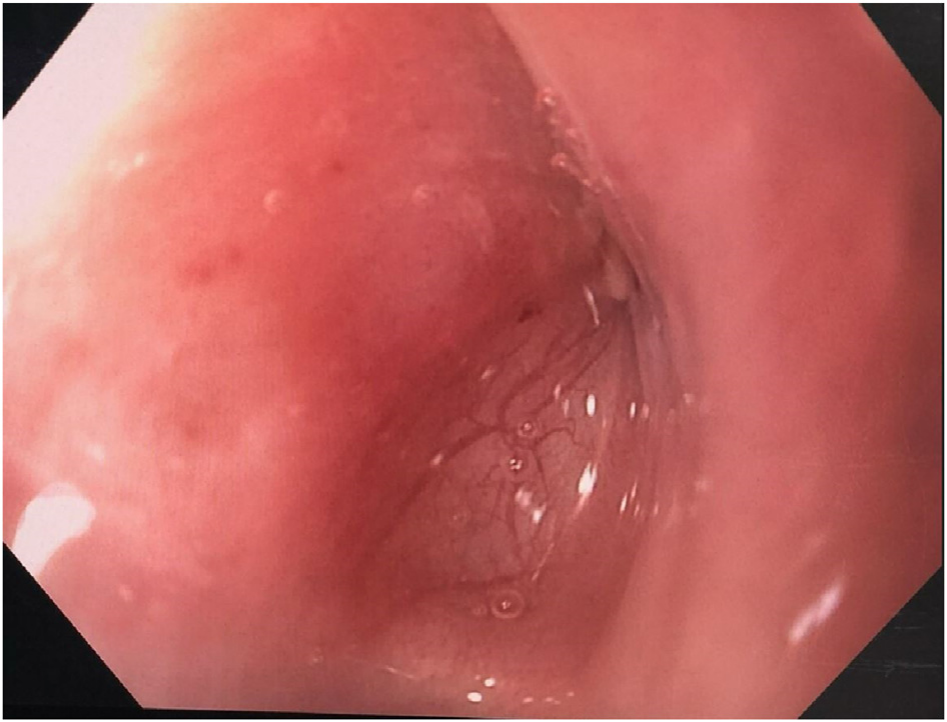
Follow-up esophagogastroduodenoscopy 4 months after discharge showing epithelialization and near-complete resolution of the esophageal defect after endoluminal wound vacuum therapy.

## Comment

AEFs represent a challenging late complication after aortic surgery. Without surgical intervention, AEF has nearly a 100% mortality rate.^2^ Traditional open operative repair often requires reoperative surgery for resection and reconstruction of the aorta in conjunction with esophageal repair or resection. This invasive approach has the potential for significant morbidity and mortality given the extensive nature of the surgery required. TEVAR has been described as a rapid, minimally invasive treatment modality to help prevent exsanguination from an aortoesophageal fistula. It has been used as both a destination therapy and as a bridge to open repair. However, TEVAR does not address the esophageal perforation that is inherent to the pathology of AEF.

To date, there have been no reports of E-Vac therapy being used to address the esophageal component of AEF. We believe that this represents an important minimally invasive option that could be used in patients who are poor candidates for open surgery. Negative pressure wound therapy using wound vacuum dressings has been used in the esophagus to treat perforation and postoperative leak with a closure rate upwards of 80%.^4^ Negative pressure wound therapy optimizes the physiology of wound healing primarily through its effects on macrodeformation, microdeformation, drainage of the wound, and by increasing circulation to the wound bed.^6^ This is of particular interest for AEF in the setting of aortic surgery because of the concerns of graft infection and mediastinal abscess formation. One limitation of E-Vac therapy for AEF is that because the aortic graft is not excised, there is potential for prolonged prosthetic graft infection. However, infection can potentially be managed with long-term suppressive antibiotics for poor operative candidates. Another concern with E-Vac therapy is the potential for significant hemorrhage. Despite several weeks of prolonged E-Vac therapy, however, the patient had no bleeding events. One other point of discussion regarding E-Vac therapy is that it can be labor-intensive. This patient required twice-weekly endoscopies for E-Vac sponge changes for 4 weeks from initiation of therapy, which is around the average time needed for an esophageal perforation to close.^4^ Fortunately, our patient tolerated these procedures and prolonged nasogastric tube placement well. Although esophageal stenting has been used as a minimally invasive treatment modality for the management of esophageal perforations, it is a poor option for the management of AEF in the setting of prior aortic surgery given the potential for pressure necrosis of the esophageal wall between the esophageal stent an aortic graft. Thus, E-Vac therapy used in conjunction with TEVAR may provide a minimally invasive treatment option for AEF in patients who are poor candidates for open surgical repair, though more data is needed to make a conclusion regarding long-term outcomes.

## Figures and Tables

**Supplemental Figure 1:**
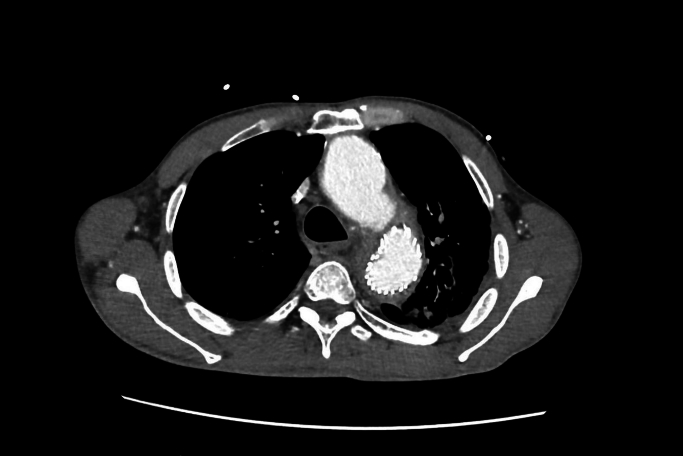


## References

[1] Yamazato T., Nakamura T., Abe N. (2018). Surgical strategy for the treatment of aortoesophageal fistula. J Thorac Cardiovasc Surg.

[2] Takeno S., Ishii H., Nanashima A., Nakamura K. (2020). Aortoesophageal fistula: review of trends in the last decade. Surg Today.

[3] Smallwood N.R., Fleshman J.W., Leeds S.G., Burdick J.S. (2016). The use of endoluminal vacuum (E-Vac) therapy in the management of upper gastrointestinal leaks and perforations. Surg Endosc.

[4] Livingstone I., Pollock L., Sgromo B., Mastoridis S. (2021). Current status of endoscopic vacuum therapy in the management of esophageal perforations and post-operative leaks. Clin Endosc.

[5] Leeds S.G., Mencio M., Ontiveros E., Ward M.A. (2019). Endoluminal vacuum therapy: how I do it. J Gastrointest Surg.

[6] Huang C., Leavitt T., Bayer L.R., Orgill D.P. (2014). Effect of negative pressure wound therapy on wound healing. Curr Probl Surg.

