# Supplementary material for: Management of an Aortoesophageal Fistula With Esophageal Endoluminal Wound Vacuum Therapy
Source: Ann Thorac Surg Short Rep. 2024 Feb 15;2(3):552–4. doi: 10.1016/j.atssr.2024.01.011 (PMC11708415; doi:10.1016/j.atssr.2024.01.011)
Supplement: Supplemental Material [file mmc1.docx]

**SUPPLEMENTAL MATERIAL**

**Supplemental Figure 1.** Follow-up chest CT prior to discharge showing no evidence of fistula tract and marked improvement of left upper lobe consolidation.
